# Weakening the subjective sensation of own hand ownership does not interfere with rapid finger movements

**DOI:** 10.1371/journal.pone.0223580

**Published:** 2019-10-04

**Authors:** Arran T. Reader, H. Henrik Ehrsson

**Affiliations:** Department of Neuroscience, Karolinska Institutet, Stockholm, Sweden; Universita degli Studi di Udine, ITALY

## Abstract

When we perform a movement we generally have a clear distinction between which parts of the world constitute our body and which parts do not. However, how the sense of ownership over our body supports movement is not yet fully understood. We aimed to see whether a sense of ownership over the hand supports the performance of rapid hand movements. In three experiments (n = 48, n = 30, n = 24), we presented participants with congruent and incongruent visuotactile and visuoproprioceptive information regarding their own hand. In keeping with previous experiments, multisensory disintegration resulted in a reduction in the subjective sensation of ownership over the hand, as reflected in questionnaire responses. Following sensory stimulation, participants were required to rapidly abduct their index finger whilst the movement was tracked. We examined the hypothesis that, should a sense of ownership over the limb be necessary for generating rapid movements with that limb, reaction time would increase when hand ownership was reduced, whilst the acceleration and velocity of the movement would decrease. We observed that reductions in own hand ownership did not interfere with rapid index finger abduction, suggesting that the motor system may not be reliant on a subjective sense of ownership over the body in order to generate movement.

## Introduction

Could we move a limb that we feel does not belong to us? Such a question may seem counterintuitive, but our ability to move might be closely tied to our sense of body ownership: the distinct sensation that our observed body belongs to us. This sensation of body ownership helps us to distinguish between our self and the surrounding environment, and is believed to arise through the integration of multisensory information, particularly co-occurring visual, tactile, and proprioceptive percepts [1,2]. By altering the congruity of these sources of multisensory information, it is possible to interfere with body ownership. The most well-known example of this is the rubber hand illusion [3], in which participants observe a false, rubber hand in a closely congruent position to their real (hidden) hand. When touches are observed on the false hand and felt on the real hand in synchrony, the participant may perceive that the rubber hand is their own. The illusion can cause the perceived position of the real hand to drift towards the fake one [4], and some individuals might also have sensations of disownership for the real hand when the illusion is induced [5–8].

The role of visual, kinaesthetic, and proprioceptive information in guiding action [9–11] suggest that body ownership and movement could be closely linked. Whilst there is evidence that illusions of body ownership can change the way in which we act upon objects in the world, previous experiments induced illusory sensations of ownership over objects external to the body that visually resembled human limbs–such as a rubber hand–by exploiting multisensory integration [12–15]. That is, by creating a scenario in which sensory cues from the body are aligned in a complementary pattern but not in keeping with veridical percepts that would accurately guide the action. For example, reaching or grasping behaviour may be influenced by inducing ownership over a hand that is different in size or posture to the veridical limb [12,13]. However, the results of these experiments, in which interaction with the environment was required, might not be in keeping with an effect of body ownership on movement *per se*. For example, ownership of a differently-sized hand might alter object-directed action through scaling of the visual environment [16–18], altering the perceived spatial relationship between the body and the world, rather than acting upon the motor system directly. As such, whilst body ownership may be important for *action*, it is still unclear whether manipulating body ownership directly interferes with the generation or performance of *movement*. Whereas sensory feedback arising from movement may contribute to changes in the sense of body ownership [19–24], and movement disorders may interfere with body ownership [25–27], it is unclear whether this effect is bidirectional. That is, could reducing the sense of ownership over a body part make it harder to move? Do we truly need to feel like a limb is ours in order to effectively move it? How might the motor system respond in this scenario?

Interestingly, there is some evidence that body illusions may influence the excitability of corticospinal motor circuits, which might suggest a role of body ownership in mediating movement. della Gatta *et al*. [28] applied single-pulse transcranial magnetic stimulation (TMS) over the primary motor cortex (M1) during the rubber hand illusion. They observed a reduction in corticospinal excitability, as measured by motor-evoked potentials (MEPs) in the first dorsal interosseous (FDI), compared to baseline in participants when they experienced synchronous but not asynchronous visuotactile stimulation of the rubber hand. Similar effects were not observed for the hand contralateral to the illusion. The authors’ explanation for the effect was that the excitability of M1 was reduced when participants disowned their own hand during the illusion. They suggested that “body ownership and the motor system are mutually interactive and both contribute to the dynamic construction of bodily self-awareness…an experimental manipulation of the sense of body ownership is accompanied by a coherent modulation of the motor system” (p. 8). However, it is still unclear whether the reduction in MEP amplitude observed by della Gatta *et al*. truly reflected disownership of the real limb. Alternatively, it could have reflected cognitive processes that are unrelated to motor control but are engaged in the complex rubber hand illusion phenomenon, e.g., the spatial recalibration of hand position sense towards the location of the model hand that occurs in this illusion (e.g., [3,29,30]), or even a physiological epiphenomenon rather than a reduction in the basic capacity of M1 to produce movement.

Other experimenters have also examined the potential influence of body ownership on corticospinal excitability, with mixed results. Kilteni *et al*. [31] observed a reduction in corticospinal excitability when participants experienced illusory amputation of their lower arm and hand. The stronger the “illusion of amputation”, as rated by the participants, the greater the reduction in corticospinal excitability. Kilteni *et al*. suggested that the illusory experience of a missing limb can influence the corticospinal motor system, as might be observed in cases of limb deafferentation, amputation, or immobilisation. Karabanov *et al*. [32], however, observed no effect of the moving rubber hand illusion [19] on corticospinal excitability, although they did observe that ownership over a rubber hand was associated with a resting inhibitory pattern of connectivity between the anterior intraparietal sulcus and M1 (see also [33]). This connectivity was not maintained in scenarios where participants had a sense of agency (a feeling of control [34]) but no ownership over the rubber hand, or no agency *and* no ownership over the rubber hand. Such effects implicate a role for body ownership in mediating the connectivity of the cortical motor system and support the idea that manipulation of the sensory signals that normally inform body ownership could feasibly influence movement. However, direct evidence for this idea is lacking. Such evidence is essential to confirm a behaviourally relevant influence of body ownership on the motor system.

Confirmation of a functional role of body ownership in movement might prove useful for the study of clinical disorders. In particular, it may benefit the development of prosthetic limbs to know if strong sensations of ownership over a prosthetic are a requirement for efficiently moving the false limb and promoting the ‘integration’ of that limb into the cortical motor system. Whilst there is evidence that amputees can perceive ownership over false limbs in the place where their real limbs used to be [35–37], it is not yet established whether these illusory sensations could ‘trick’ the motor system into fully integrating a replacement for the missing limb (but see [38]). In addition, clinical disorders in which patients deny ownership of a limb [39–42] frequently co-occur with hemiplegia. Understanding how sensations of limb *disownership* influence movement could feasibly point the way to more personalised rehabilitation protocols for these patients (see [43] for a broader discussion in this context). Experimentally manipulating the sense of ownership over the real body, without inducing ownership over a false limb, could be particularly informative in this context, because the phenomenology and multisensory mechanisms would arguably be more similar to that of neurological patients denying limb ownership [44–47]. Furthermore, the influence of body ownership on the cortical motor system described by aforementioned experimental articles should not only have functional relevance, but should also be evident in paradigms other than the rubber hand illusion. Previous research suggests that presenting individuals with incongruent multisensory information regarding their real body can reduce the subjective sensation of ownership over the body or reduce skin conductance response to threats [48–54], suggesting that the real body can be both explicitly and implicitly disowned in experimental settings, without ‘replacing’ it with a false limb. This might provide a more pure way of examining the role of body ownership in the movement of the real body. This is important since it is not yet clear whether sensations of real hand disownership during the rubber hand illusion are phenomenologically similar to those that can be observed in multisensory disintegration, or if they can even be consistently induced [8,55,56]. With this in mind, we planned the following experiment.

Motor output for simple movements is closely tied to the excitability of the motor system [57–63]. Therefore, if limb disownership downregulates the motor system [28], then reducing the perceived ownership of a participant’s real seen hand should interfere with the movement of that hand during a simple reaction time (RT) task. To test this, we assessed rapid finger movements following multisensory disintegration of signals from the hand caused by exposure to incongruent visual, tactile, and proprioceptive information in a setup using online digital manipulation of visual feedback (see below). We tested a rapid abduction movement of the right index finger because such movement requires the activation of only a few muscles, including the right FDI, and thus probes the corticospinal system’s capacity to produce basic movement. We expected increases in abduction RT and decreases in the movement peak acceleration (PA) and peak velocity (PV) following a reduction in the sensation of ownership over one’s own hand.

## Experiment 1

### Methods

#### Participants

We recruited 48 participants (mean±SD age = 27.0±5.02 years, 23 female, 6 left-handed (self-report)) from Karolinska Institutet and the surrounding area. This sample size was based on the need to counterbalance the four conditions used in the experiment and deemed suitable given the effect size of dz = 0.74 reported by della Gatta *et al*. [28] for the reduction in MEP amplitude following the rubber hand illusion compared to baseline: a power analysis in G*Power 3.1 [64] for a two-tailed t-test at 95% power with dz = 0.74 resulted in a recommended sample size of 26. The experiment was approved by the Regional Ethical Review Board of Stockholm (ref: 2010/548-31/2 and 2018/322), and participants provided written informed consent.

#### Materials and stimuli

**Fig 1:**
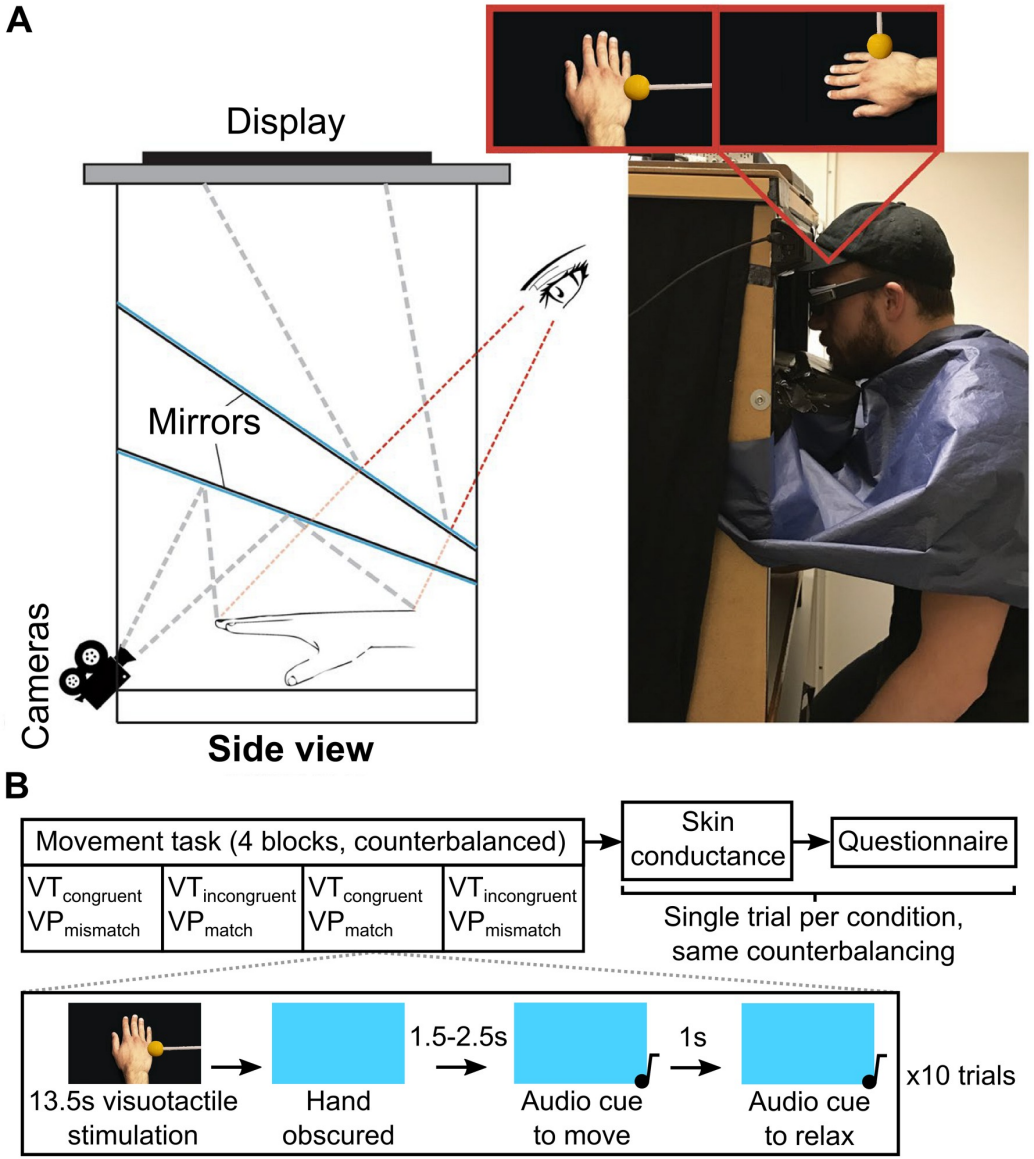
Hand illusion box and experiment 1 procedure. A) Hand illusion box. The two possible seen orientations of the real hand are highlighted in red (left: VP_match_, right: VP_mismatch_). Adapted with permission from Abdulkarim & Ehrsson [65]. B) Procedure for experiment 1. Participants took part in three tasks, each one assessing four conditions in a counterbalanced order. In the movement task, conditions were performed in blocks of 10 trials, with each trial featuring a single movement without visual feedback (a blue screen obscured the hand image) following a 13.5s visuotactile stimulation period during which participants passively observed the hand being stroked. The movement was a single speeded abduction of the right index finger in response to an auditory cue. In the skin conductance and questionnaire tasks, each condition was assessed using a single trial in the same counterbalanced order, where the knife threat or the questionnaire statements were presented after each 13.5s visuotactile stimulation period.

A custom visual feedback setup (the ‘hand illusion box’ previously reported by Abdulkarim & Ehrsson [65], developed by Martti Mercurio and Andreas Kalckert) was used to provide participants with 3D information regarding their hand (Fig 1A). The hand illusion box is a 76x77.5x51 cm box, open at two ends, containing a series of angled mirrors. Two cameras (AVT Guppy Pro, Stadtroda, Germany) are used to relay live visual information, recorded at 60 frames per second, regarding the hand placed in the box. This visual information is displayed on a 3D compatible monitor (ASUS, Taipei, Republic of China), which can be observed using 3D glasses in combination with a 3D sensor (NVIDIA, California, USA). The mean±SD delay between camera capture and display is approximately 65.1±3.25 ms, and the visual information is displayed on the monitor at 120 Hz (60 Hz for each camera). The image of the hand is displayed at an angle matching that of natural top-down viewing (i.e., from the first-person perspective). For this experiment, the image on the screen could be rotated 90° counter-clockwise to create a visuoproprioceptive conflict and/or delayed by 1.25 seconds to create a visuotactile conflict when tactile stimulation was applied (detailed below). A cloth obscured the participants’ body and arms from the shoulder down.

The position of the participants’ right index finger was recorded using a wired Polhemus Fastrak motion-tracking system (Polhemus Inc., Colchester, VT, USA) at 120 Hz with 6 degrees of freedom (x, y, z, azimuth, elevation, and roll). The tracker was placed over the fingernail and attached using adhesive medical tape. The motion-tracking transmitter was placed inside the hand illusion box, out of the view of the participant. Data were collected using MATLAB R2017b (Mathworks, Inc.) and the HandLabToolBox (https://github.com/TheHandLab/HandLabToolBox).

Participants responded to seven questionnaire items adapted from Gentile *et al*. [48] and della Gatta *et al*. [28], addressing the perception of body (dis)ownership (Table 1). Responses to the statements were given using a 7-point Likert scale ranging from -3 (“strongly disagree”) to +3 (“strongly agree”).

**Table 1:** Questionnaire items. Statements were adapted from previous studies [28,48].

Item	Statement	Purpose
**S1**	“It felt like my own real hand was located where I saw it”	Assess visuoproprioceptive integration
**S2**	“It felt as if the touch I experienced on my hand was directly caused by the object I saw”	Assess visuotactile integration
**S3**	“It felt as if I was looking at my own hand”	Assess hand (dis)ownership
**S4**	“It seemed like I was unable to move my hand”	Assess possible loss of agency over the hand
**S5**	“It seemed like my hand had disappeared”	Control statement for task demands and suggestibility
**S6**	“When I saw the object, I had the sensation that my hand was numb”	Control statement for task demands and suggestibility
**S7**	“I did not know exactly where my hand was located”	Control statement for task demands and suggestibility

To record an objective measurement of limb ownership [48], skin conductance response to threat was recorded using a Biopac MP150 data acquisition system (Biopac Systems, Inc., Goleta, USA). Data were collected at 100 Hz using Biopac software AcqKnowledge 3.9.1 from two electrodes attached to the participant’s left index and middle fingertips. Skin conductance response to threat was induced through the presentation of a blunted but sharp-looking kitchen knife. The knife was 29 cm in length (blade 16 cm), with a plastic blue handle. The blade was coated with tape to ensure that it was no longer sharp.

During the experiment participants wore earplugs to reduce distraction and headphones (Maxell, Tokyo, Japan) to provide them with audio triggers indicating when to move their index finger. An experimenter applied tactile stimulation to the hand using a custom-built probe: a bright yellow compressed cotton ball on the end of an L-shaped white stick.

#### Procedure

Participants stood or sat at the illusion box, depending on which position was most comfortable and allowed them to fully relax their right arm and hand. The image of their right hand was increased or decreased in size by the experimenter prior to the start of the experiment to be as close to actual width and length as possible. This was done to account for the fact that thicker hands would result in a larger image on the screen, since the top of the hand was closer to the mirror. The mean±SD size of the hand on the screen was approximately 85.4±7.00% of the actual length and 95.1±8.80% of the actual width, which we judged sufficiently close to real hand size for illusion induction. Participants were asked to observe their hand, keep it relaxed, and avoid moving it except when explicitly required by the task.

Participants first took part in four blocks of ten trials to motion-track rapid index finger abduction following visuotactile stimulation (Fig 1B). A single condition was tested in each block, with each condition assessing a different combination of visuotactile (VT) or visuoproprioceptive (VP) conflict: VT_congruent_VP_match_ (the hand is seen as in reality, with no multisensory disintegration), VT_incongruent_VP_match_ (visual feedback is delayed by 1.25 seconds, causing visuotactile conflict), VT_congruent_VP_mismatch_ (the image of the hand is rotated 90° counter-clockwise, causing visuoproprioceptive conflict and strongly violating the spatial constraints of body ownership [1,2,29,66]), VT_incongruent_VP_mismatch_ (visual feedback is delayed by 1.25 seconds and the image of the hand is rotated 90° counter-clockwise). We expected that feelings of ownership for the hand (questionnaire statement S3) would decrease in the following order, as reported by Gentile *et al*. [48]: VT_congruent_VP_match_, VT_incongruent_VP_match_, VT_congruent_VP_mismatch_, VT_incongruent_VP_mismatch_.

In each trial within a block, visuotactile stimulation occurred for a period of 13.5 seconds, during which participants passively observed their right hand. This stimulation consisted of six strokes, 1 second each in duration, over the hand using the probe. Strokes were performed over the top of the hand, just below the metacarpophalangeal joint, in a medial to lateral direction. There was a 1.5 second period without tactile stimulation between each touch. These stimulation parameters, along with the 1.25 second delay described above, have previously been used to effectively reduce the sensation of ownership over the real hand in a multisensory disintegration paradigm similar to the current one [48].

Following the 13.5 second visuotactile stimulation period, the image of the hand disappeared from the screen to be replaced with a blue background (initiated by the experimenter). Following an arbitrarily chosen randomised interval of 1.5 to 2.5 seconds (during which the blue screen was present), a high-pitched tone played in the headphones, indicating that the participant should abduct their right index finger as quickly as possible, and keep it abducted until they heard a second, lower pitch tone play, 1 second later. They then relaxed their finger, the image of their hand reappeared on the screen, and the next trial began (with a new 13.5 second visuotactile stimulation period). Vision of the hand was obscured during movement to avoid explicit movement-outcome breakdown, since corticospinal excitability may vary depending on the temporal congruence of performed and observed movement [67]. That is, in the VT_incongruent_ conditions, we did not want to dilute any effects of limb disownership by introducing a scenario in which participants would observe their finger moving with a delay, potentially interfering with the sensations of ownership and agency in later trials. The hand was therefore obscured in all conditions for consistency. Furthermore, at any point in the experiment in which the visuotactile or visuoproprioceptive condition changed (i.e., between blocks), the blue screen was presented to ensure that the conditions were visually distinct and that participants did not observe the image suddenly rotate or delay.

Following collection of the kinematic data, participants underwent an additional trial in each condition (in their counterbalanced order) to assess subjective (questionnaire) and objective (skin conductance) responses to the illusion. Skin conductance data were collected first. Participants observed the visuotactile stimulation for 13.5 seconds in each condition, after which the experimenter triggered a marker on the skin conductance trace by pressing a button, then rapidly approached the participant’s hand with the knife. The knife was presented above the hand from a lateral direction for a period of approximately 1 second and then withdrawn. Questionnaire data were collected in the same fashion, with participants observing a single period of visuotactile stimulation for each of the 4 conditions. Following 13.5 seconds of visuotactile stimulation, the screen turned blue, and the experimenter verbally presented the participants with the statements in a random order, recording their responses on a computer for later analysis. Questionnaire data were collected at the very end of the session, to avoid biasing participant behaviour in previous elements of the experiment.

#### Data analysis

Raw and processed data are available from the Open Science Framework (https://osf.io/zdb8e).

Each questionnaire item was compared between conditions using Wilcoxon signed-rank tests in SPSS 21, using a FDR-corrected alpha threshold [68] across all comparisons. The skin conductance response to threat was assessed by subtracting the minimum value of skin conductance (in μS) during the 5 seconds prior to the threat from the maximum skin conductance during the 10 seconds following threat. Thus, we computed the amplitude of the skin conductance response for each condition. These relatively long data selection periods were chosen to account for the time between the experimenter trigger and knife presentation, and the delayed video feedback presented in the VT_incongruent_ conditions. The examination of skin conductance data during analysis confirmed that this approach was suitable. One participant was excluded from the skin conductance analysis due to technical issues with data collection, and participants with a skin conductance response <0.01 μS in any condition were excluded from the analysis of skin conductance data, leaving 17 participants for analysis.

Skin conductance response was analysed between conditions using two-tailed paired samples t-tests in JASP 0.9 [69] using an FDR-corrected alpha threshold. We also performed Bayesian paired samples t-tests in JASP [70], using a default Cauchy prior with a scale of 0.707, zero-centred (where 50% of the density is located between effect sizes -0.707 and 0.707, for a two-sided hypothesis). This default prior distribution effectively specifies an alternative hypothesis in which one is 50% confident that the true effect size lies between -0.707 and 0.707. This analysis provided information regarding the level of support for the null hypothesis (condition A = condition B) compared to the alternative hypothesis, or for the alternative hypothesis (condition A ≠ condition B) compared to the null hypothesis, given the data. We used a typical heuristic for assessing evidence for either hypothesis, in which BF_10_>3 suggests better support for the alternative hypothesis, and BF_10_<0.333 better support for the null hypothesis [71].

To assess the link between subjective and objective measurements of hand ownership, we correlated participant skin conductance response amplitude in each condition with their responses to S3 using Kendall rank correlation (tau-b) with an FDR-corrected alpha threshold, along with a Bayesian Kendall rank correlation (stretched beta prior width of 1) [72].

A custom automated script in MATLAB R2017b, along with kinematic data pre-processing modules from the HandLabToolBox were used for pre-processing and extraction of kinematic variables. The analysis routines processed the position data from each trial of each participant and rejected artefacts (e.g., trials with missing samples or spikes resulting from electromagnetic interference). Single timepoint spikes (>3 SD from the mean) in each trial’s double-differentiated time series were deemed electromagnetic artefacts and removed by interpolation across three adjacent samples on either side. The position data were also filtered using a second-order dual-pass Butterworth filter with a 10 Hz low-pass cut-off. Trials in which the RT was shorter than 200 ms or longer than 600 ms, in which the PV was lower than 10 cm/s or greater than 100 cm/s, or in which the participant did not stop moving before the tone signalling them to return their finger back to a resting position, were excluded. All trials were then visually inspected for artefacts, and to check if participants performed a second finger movement during the movement period with faster velocity than the initial movement (suggesting that they moved their finger back to the starting position prior to the end of the movement period). Trials were also excluded in those cases. A total of 89.7% of trials were maintained for statistical analysis.

RT was measured as the time between the presentation of the tone that indicated the participant to move, and when the tracker 3D velocity reached an arbitrary value of 5 cm/s. Movement end time was considered the point at which the tracker 3D velocity returned below 5 cm/s, and the 3D PA and 3D PV were extracted from between the RT and this timepoint.

Differences between RT, PA, and PV across each condition were assessed using Bayesian paired samples t-tests, and two-tailed paired samples t-tests with an FDR-corrected alpha threshold for all kinematic comparisons. We hypothesised that, should limb disownership interfere with the motor system, we would observe a statistically significant increase in RT and decreases in PA and PV in scenarios with a greater visuotactile and visuoproprioceptive mismatch. In particular, we expected that RT would increase and PA/PV decrease in the following order: VT_congruent_VP_match_, VT_incongruent_VP_match_, VT_congruent_VP_mismatch_, VT_incongruent_VP_mismatch_.

To examine the effects of limb disownership sensations on kinematics at the individual level we correlated the kinematic variables with responses to S3 in each condition, predicting that those with a greater reduction in the sensation of ownership would move with a greater RT and smaller PV and PA. These analyses were performed with Kendall rank correlations (tau-b) with an FDR-corrected alpha threshold, along with Bayesian Kendall rank correlations (stretched beta prior width of 1).

In all default Bayesian analyses, we also assessed the robustness of the Bayes factor. That is, we report the maximum Bayes factor for a given distribution and the associated scaling value of the prior (i.e., the Cauchy prior width or stretched beta prior width) in that instance.

### Results

#### Questionnaire

**Fig 2:**
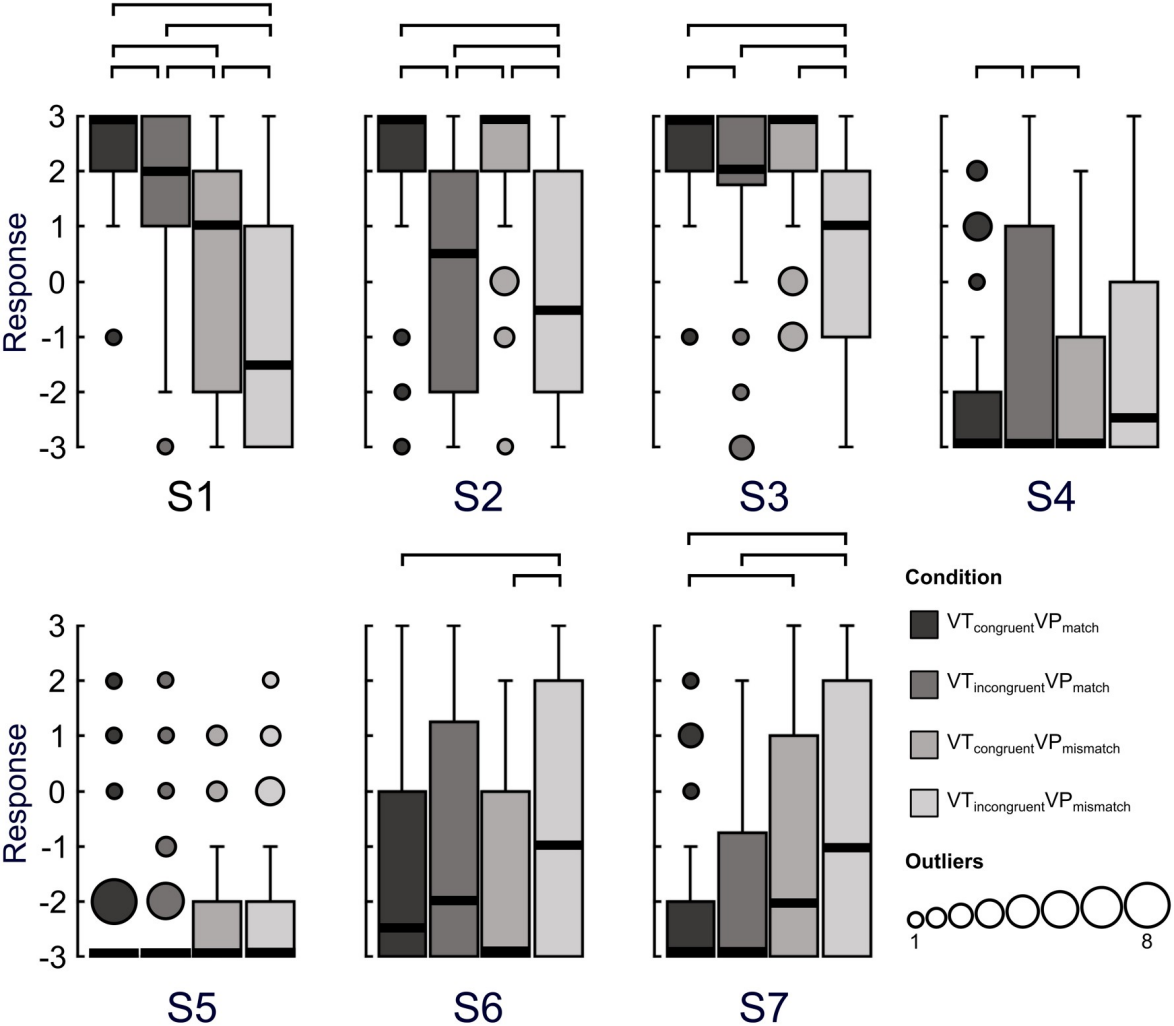
Experiment 1 questionnaire results. Subjective experience during the four levels of multisensory disintegration was assessed by comparing agreement with questionnaire statements. Boxplots indicate group responses to each statement for each condition (-3 = strongly disagree, +3 = strongly agree. The solid black lines indicate the median. The number of outliers at any given response value are indicated by the size of the outlying point. The connections above the boxes indicate statistically significant differences between conditions (Wilcoxon signed-rank test, FDR-corrected, p≤.00488).

We found that participants showed a reduction in perceived ownership over their own hand as multisensory signals were broken down, indicated by the responses to S3 (Fig 2). In keeping with our hypothesis, participants were less inclined to agree with the statement “it felt as if I was looking at my own hand” in the VT_incongruent_VP_mismatch_ condition compared to the VT_congruent_VP_match_ (Z = -4.80, p<.001), VT_incongruent_VP_match_ (Z = -3.83, p<.001), or VT_congruent_VP_mismatch_ (-4.15, p<.001) conditions. In addition, we also observed a small but statistically significant reduction in perceived ownership over the hand in the VT_incongruent_VP_match_ compared to VT_congruent_VP_match_ condition (Z = -2.85, p = .00436), suggesting that visuotactile incongruence alone was strong enough to induce a reduction in the subjective sensation of ownership over the hand.

The analysis of S1 indicated that participants felt less like their hand was located in the position shown on the screen as visuotactile and visuoproprioceptive stimuli disintegrated. Agreement with the statement “it felt like my own real hand was located where I saw it” was statistically significantly reduced compared to the VT_congruent_VP_match_ condition in the VT_incongruent_VP_match_ (Z = -3.13, p = .00175), VT_congruent_VP_mismatch_ (Z = -4.87, p<.001), and VT_incongruent_VP_mismatch_ (Z = -5.43, p<.001). Agreement was reduced compared to the VT_incongruent_VP_match_ condition in the VT_congruent_VP_mismatch_ (Z = -3.04, p = .00235) and VT_incongruent_VP_mismatch_ (Z = -4.85, p<.001) conditions. Agreement was also reduced in the VT_incongruent_VP_mismatch_ condition compared to VT_congruent_VP_mismatch_ (Z = -2.90, p = .00374) condition.

Responses to S2 suggested that visuotactile incongruence induced a reduction in the perceptual binding of visual and tactile events on the hand. In particular, participants felt less like the touch they experienced was caused by the probe they saw in the VT_incongruent_VP_mismatch_ condition compared to the VT_congruent_VP_match_ (Z = -4.87, p<.001), VT_incongruent_VP_match_ (Z = -2.94, p = .00329), and the VT_congruent_VP_mismatch_ (Z = -5.25, p<.001) conditions. A similar reduction was observed for the VT_incongruent_VP_match_ condition compared to the VT_congruent_VP_match_ (Z = -4.51, p<.001) and VT_congruent_VP_mismatch_ (Z = -4.87, p<.001) conditions.

The greatest diminishing effect on the subjective sense of agency over the hand was found when participants observed their hand under incongruent visuotactile stimulation, but in a matched proprioceptive position. That is, agreement to statement S4 (“It seemed like I was unable to move my hand”) increased in the VT_incongruent_VP_match_ condition compared to the VT_congruent_VP_match_ (Z = -3.07, p = .00217) and VT_congruent_VP_mismatch_ (Z = -2.82, p = .00488) conditions, although the median value remained at -3, indicating that many participants still strongly rejected this statement even in the former condition.

Participants showed some reduced disagreement to S6, a control statement assessing the degree of affirmation of numbness sensations in the hand, in the VT_incongruent_VP_mismatch_ condition when compared to VT_congruent_VP_match_ (Z = -2.92, p = .00354) and VT_congruent_VP_mismatch_ (Z = -3.20, p = .00140). The relatively short duration of visuotactile stimulation is one possible explanation for this result. That is, if participants experience incongruent visuotactile stimulation for a limited time, without a reduction in the sensation of own hand ownership, they may perceive this experience as numbness–the observation of touch on the hand without a concomitant tactile sensation.

Participants also showed reduced disagreement (though generally not agreement) to S7, a control statement querying knowledge of the location of the hand, in the VT_incongruent_VP_mismatch_ condition compared to the VT_incongruent_VP_match_ (-3.15, p = .00166) and VT_congruent_VP_match_ (-4.41, p<.001) conditions. Participants also showed reduced disagreement in the VT_congruent_VP_mismatch_ condition versus VT_congruent_VP_match_ (Z = -3.62, p<.001) condition.

No statistically significant effects were observed across conditions for control statement S5 (Table A in S1 File). All other non-significant effects are also reported in Table A.

#### Skin conductance response

Whilst there were no FDR-corrected statistically significant differences between skin conductance response across the four conditions, a Bayesian analysis did suggest that there was some evidence for differences between conditions. Namely, the amplitude of the skin conductance response to threat was lower in the VT_incongruent_VP_match_ condition (mean±SE = 0.459±0.0840 μS) than the VT_congruent_VP_match_ (0.842±0.156 μS; t(16) = -2.77, p = .0137, g_rm_ = 0.660, BF_10_ = 4.14, max BF_10_ = 4.29 at Cauchy prior width 0.508) and VT_incongruent_VP_mismatch_ (0.924±0.194 μS; t(16) = -3.08, p = .00717, g_rm_ = 0.572, BF_10_ = 7.09, max BF_10_ = 7.16 at Cauchy prior width 0.598) conditions. This suggested that the greatest reduction in skin conductance response to threat was observed in the VT_incongruent_VP_match_ condition and not in the VT_incongruent_VP_mismatch_ condition, as predicted.

There was no statistically significant difference between the VT_congruent_VP_match_ condition and the VT_congruent_VP_mismatch_ (0.775±0.175 μS; t(16) = 0.398, p = .696, g_rm_ = 0.0931, BF_10_ = 0.267, max BF_10_ = 1.00 at Cauchy prior width 0.0005) condition. There were also no significant differences between the VT_congruent_VP_match_ condition and the VT_incongruent_VP_mismatch_ (t(16) = -0.370, p = .716, g_rm_ = 0.107, BF_10_ = 0.265, max BF_10_ = 1.00 at Cauchy prior width 0.0005) condition, between the VT_incongruent_VP_match_ and VT_congruent_VP_mismatch_ (t(16) = -1.93, p = .0713, g_rm_ = 0.499, BF_10_ = 1.12, max BF_10_ = 1.42 at Cauchy prior width 0.245) conditions, or between the VT_congruent_VP_mismatch_ and VT_incongruent_VP_mismatch_ (t(16) = -0.684, p = .504, g_rm_ = 0.186, BF_10_ = 0.306, max BF_10_ = 1.00 at Cauchy prior width 0.0005) conditions.

There were no statistically significant correlations between S3 and the amplitude of skin conductance response for any of the four conditions (|r_τ_|≤.307, p≥.101; Table B in S1 File). The Bayesian analyses did not result in convincing support for either the null or experimental hypothesis in any case (0.339≤BF_10_≤1.22, max BF_10_ = 1.56 at stretched beta prior width 0.257). Therefore, we could not conclusively determine whether participants with a greater reduction in the subjective sense of ownership over their hand showed a smaller skin conductance response to threat towards that hand.

#### Kinematics

**Fig 3:**
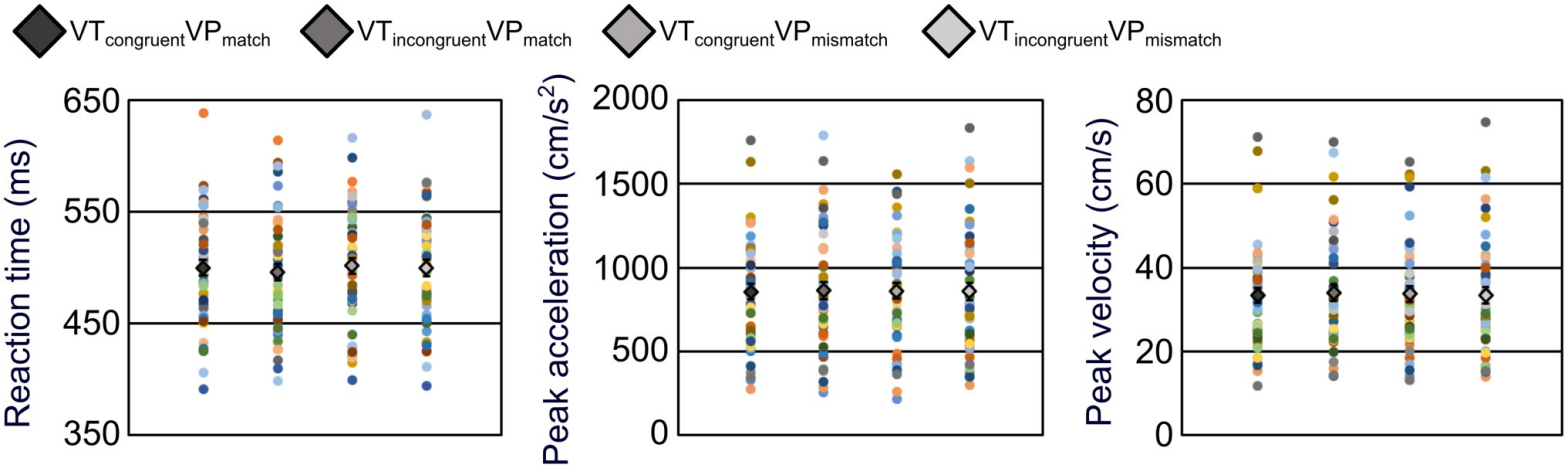
Experiment 1 kinematic results. The reaction time, velocity, and acceleration of rapid index finger abduction were compared across four levels of multisensory disintegration. Diamonds indicate mean values with between-subjects standard error bars. The coloured circles show individual participant values.

There were no statistically significant differences between any of the conditions for any of the kinematic parameters (Fig 3). In all cases, |t(47)|≤1.30, p≥.201, 0.157≤BF_10_≤0.343, and max BF_10_ = 1.00 at Cauchy prior width 0.0005. This suggested that the null hypothesis was more likely than an alternative hypothesis (Tables C-E in S1 File), except for very small prior widths highlighted in the robustness analysis, where the hypotheses were deemed equally likely. Thus, RT, PA, and PV appeared to be unaffected by multisensory disintegration.

Additionally, there were no statistically significant FDR-corrected correlations between kinematic variables and responses to S3 (|r_τ_|≤.227, p≥.0326; Table F in S1 File). In most cases, the Bayesian analysis suggested more support for the null hypothesis than the alternative hypothesis (0.187≤BF_10_≤2.36). These correlation results suggested little evidence in favour of an effect of reduced hand ownership on rapid finger movement. Whilst we did observe correlations with a p-value below .05 between PA and S3 (r_τ_ = .220, p = .0390, BF_10_ = 2.00, max BF_10_ = 3.14 at a stretched beta prior width of 0.171), and PV and S3 (r_τ_ = .227, p = .0326, BF_10_ = 2.36, max BF_10_ = 3.61 at a stretched beta prior width of 0.198) in the VT_incongruent_VP_mismatch_ condition, possibly suggesting that those who felt less ownership over their own hand tended to move their finger more slowly and with smaller acceleration, these effects were not statistically significant at an FDR-corrected threshold, and the Bayes factor only provided support for the alternative hypothesis with a prior specifying a considerably larger proportion of the density surrounding zero.

Since we observed statistically significant differences in S4, assessing the sense of agency, and manipulations of agency may also influence the motor system [54,67], we decided post hoc to correlate kinematic parameters with responses to this statement (Table G in S1 File). However, as with S3, there were no statistically significant correlations between kinematic variables and responses to S4 (|r_τ_|≤.214, p≥.0552), and in most cases the Bayesian analysis suggested support for the null hypothesis (0.188≤BF_10_≤1.78, max BF_10_ = 2.86 at a stretched beta prior width of 0.171).

#### Post hoc assessment of the illusion-susceptible subset

To assess whether the general lack of an effect of hand disownership on kinematics was truly convincing, we examined only the subset of participants (n = 34) who experienced a reduction in the subjective sensation of hand ownership in the VT_incongruent_VP_mismatch_ condition, as defined by a reduced response to S3 when compared to the VT_congruent_VP_match_ condition. We compared their kinematics in the VT_congruent_VP_match_ and VT_incongruent_VP_mismatch_ conditions with one-sided Bayesian paired samples t-tests, in the direction of our original hypotheses (that weakened hand ownership would increase RT and decrease PA and PV). We used a normally distributed prior centred on two different effect sizes.

We based these effect sizes on the results of della Gatta *et al*. [28]. This was the most relevant previous experiment, since they suggested that own limb disownership (in the context of the rubber hand illusion) may downregulate the excitability of the corticospinal motor circuit involved in controlling that limb. They observed that MEPs were reduced in the synchronous condition of the rubber hand illusion compared to baseline. The effect size in this comparison was dz = 0.74. We reasoned that since externally induced MEPs (i.e., through a magnetic pulse applied over M1) could result in a larger effect than might be observed in naturalistic movement, our second prior was centred on half this standardised effect size (0.37). The SD of the prior distributions was set at 0.19, such that the medians of the two alternate effect sizes were separated by approximately 2 SDs. To better establish how convincing the Bayesian output was, we also compared conditions using the default Cauchy prior width of 0.707, and assessed the robustness of the Bayes factor to different (one-sided, zero-centred) Cauchy prior widths, reporting the maximum Bayes factor and associated prior width.

When comparing the VT_congruent_VP_match_ condition with the VT_incongruent_VP_mismatch_ condition, an increased RT following reduced ownership over the hand was observed in only 38.2% of participants. Decreased PA and PV were observed in 52.9% and 50% of participants, respectively. The effect size centred on 0.74 suggested greater support for the null hypothesis than the alternative hypothesis for RT (mean±SE = 499±8.71 ms versus 493±8.00 ms, BF_10_ = 0.00111, g_rm_ = 0.129), PA (841±53.3 cm/s^2^ versus 836±61.3 cm/s^2^, BF_10_ = 0.0153, g_rm_ = 0.0147), and PV (32.2±2.00 cm/s versus 32.0±2.26 cm/s, BF_10_ = 0.092, g_rm_ = 0.0161). The effect size centred on 0.37 also suggested support for the null hypothesis for RT (BF_10_ = 0.0645), PA (BF_10_ = 0.276), and PV (BF_10_ = 0.284). The default Cauchy prior suggested support for the null hypothesis for RT (BF_10_ = 0.0814, max BF_10_ = 0.985 at Cauchy prior width 0.0005), PA (BF_10_ = 0.216, max BF_10_ = 1.00 at Cauchy prior width 0.0005), and PV (BF_10_ = 0.220, max BF_10_ = 1.00 at Cauchy prior width 0.0005). These results suggested that a reduced feeling of ownership over one’s own hand was not associated with an increased RT or decreased PA and PV.

### Discussion

In keeping with previous findings [15,48–54], we observed that the disintegration of visual, tactile and proprioceptive signals from participants’ own hand resulted in a reduction in perceived ownership over that hand (S3). This was observed for both VT_incongruent_VP_match_ and VT_incongruent_VP_mismatch_ conditions. However, there was little evidence that any condition was associated with changes in rapid movement performance. In fact, comparisons of the kinematic parameters indicated that the null hypothesis was more likely than an alternative hypothesis (BF_10_<0.333) in almost every case, both at the group and individual level (correlation with S3). Although there were some statistical trends suggesting that participants with a smaller subjective sensation of ownership in the VT_incongruent_VP_match_ condition had a reduced PA and PV for finger movements, this was not observed when explicitly testing our hypotheses in only those participants who reported a reduction in ownership for their hand. Taken together, the results from this experiment suggest that a reduced sensation of ownership over the hand is unlikely to induce changes in rapid movement performance.

Regarding our objective measure of hand disownership, we observed reductions in the skin conductance response to threat only in the VT_incongruent_VP_match_ condition and not in the VT_incongruent_VP_mismatch_ as we had expected. Moreover, we found no statistically significant correlations between the skin conductance response to threat and responses to S3. The predictable nature of the threat presentation in the present study was probably responsible for this, as the knife threat was always presented at the same time in a way that the participant could learn to expect. Anecdotally, many participants showed reductions in the size of the skin conductance response to threat with each additional threat presentation, which indicates substantial attenuation effects in the data. Moreover, only 17 out of 40 participants demonstrated reliable skin conductance response in all conditions and were included in the analysis. Thus, the skin conductance response data were inconclusive with respect to the hypothesis we were testing, and the analysis may have been underpowered to reliably detect a correlation between skin conductance response and S3.

Although we observed statistically significant reductions in the sense of ownership of the hand as assessed with questionnaire statement S3, the absolute rating scores were relatively high, with median scores indicating that many participants were still affirming ownership of the hand in view rather than explicitly denying ownership. We speculate that the ratings could have also been influenced by recognition-related rather than ownership-related aspects of perception, such that participants possibly understood the statement to regard the degree to which they believed the hand on the screen *looked* like their hand, rather than *felt* like their hand. This would have reduced the likelihood of observing negative ratings on S3, i.e., explicit feelings of disownership for the hand.

We decided to run a subsequent experiment with a modified version of our paradigm to confirm or refute the lack of an effect of reduced hand ownership on the performance of rapid finger movements. We aimed to increase the intensity of multisensory disintegration, add other subjective assessments for disownership, and provide a less predictable protocol for presenting the threat stimuli for the skin conductance recordings.

## Experiment 2

### Methods

Unless otherwise stated, the methods were identical to experiment 1.

#### Participants

We recruited 30 new participants (mean±SD age = 28.4±5.96 years, 17 female, 2 left-handed). None had taken part in the previous experiment. A smaller sample size was used given the expectation that our new paradigm would increase the strength of disownership sensations. This sample size was still in keeping with the recommended sample size based on della Gatta *et al*. [28] as highlighted in experiment 1. The experiment was approved by the local ethics committee (ref: 2010/548-31/2 and 2018/322), and participants provided written informed consent.

#### Materials and stimuli

We added an additional four statements to the questionnaire presented to participants (Table 2) to better dissociate recognition (S8) and (dis)ownership of the hand (S9, S10, S11).

**Table 2:** Additional questionnaire items.

Item	Statement	Purpose
**S8**	“The hand I saw looked like my own hand”	Assess visual recognition of the hand as one’s own
**S9**	“The hand I saw felt like my own hand”	Assess hand ownership
**S10**	“It didn’t feel like the hand I saw was my own”	Assess hand disownership
**S11**	“It felt like the hand I saw didn’t belong to me”	Assess hand disownership

#### Procedure

In this experiment, there were only two conditions: VT_congruent_VP_match_ and VT_incongruent_VP_mismatch_. A number of steps were taken to increase the potential for disownership sensations. The stroking of the hand with the probe was performed in the following way, with each movement lasting 1.25 seconds: over the top of the hand in a medial to lateral direction, down the lateral side of the hand and little finger, pause, over the top of the hand in a lateral to medial direction, down the medial side of the index finger, pause. The visual delay in the VT_incongruent_VP_mismatch_ condition was 2.5 seconds, meaning that visuotactile incongruence could be spatial as well as temporal. The duration of visuotactile stimulation was also increased to 30 seconds, such that each pattern of strokes was repeated 4 times. Based on previous studies [48,73], we reasoned that longer trials in combination with combined temporal and spatial visuotactile incongruence should enhance the disownership effect relative to our first experiment. Participants took part in two blocks of each condition in a counterbalanced order (ABAB or BABA). In each block there were 5 trials. The mean±SD size of the hand on the screen was approximately 89.4±5.33% of the actual length and 103±5.80% of the actual width.

To maximise the possibility of detecting kinematic effects following illusion induction, we also reduced the maximum possible duration between visuotactile stimulation and the randomised response tone. In this experiment, participants were cued to respond at a random time between 0.5 and 1 second following the blue screen obscuring vision of the hand.

The electrodes for recording skin conductance were attached to the participants’ left hand at the start of the experiment, and an extra period of tactile stimulation was randomly inserted between two of the finger movement trials in each block. In this additional period, the screen did not turn blue after visuotactile stimulation, but instead, the experimenter rapidly approached the participant’s hand with a knife, as in experiment 1, and the skin conductance response was registered before starting the subsequent trial. This resulted in two skin conductance response values for both experimental conditions.

Following motion-tracking and skin conductance recording, participants took part in an additional trial for each condition (in the same counterbalanced order); after each trial, the screen turned blue and the participants were verbally presented with the 11 questionnaire statements in a random order.

#### Data analysis

Questionnaire statements were compared between conditions using Wilcoxon signed-rank tests with an FDR-corrected alpha threshold. The skin conductance response to threat for each condition was taken as the mean response to the two knife presentations. Responses <0.01 μS were not included in the mean value, and participants were excluded from the analysis of skin conductance response if both responses to either condition were <0.01 μS. Twenty-eight participants were maintained for any analysis examining the skin conductance response to threat. The skin conductance response to threat in the two conditions were compared using a two-tailed paired t-test and a Bayesian paired samples t-test (Cauchy prior of 0.707, zero-centred).

To assess the relationship between subjective and objective measures of hand ownership, we correlated participant responses to S3, S9, S10, and S11 with the skin conductance response to threat in each condition. This was performed with Kendall rank correlations, using an FDR-corrected alpha threshold, along with Bayesian Kendall rank correlations (stretched beta prior width of 1).

Kinematic data pre-processing was performed as in experiment 1. For two participants, the first VT_congruent_VP_match_ block was not recorded due to technical issues. Following exclusions, 84.2% of trials were maintained for statistical analysis. Two-tailed paired t-tests were used to compare RT, PA, and PV between the VT_congruent_VP_match_ and VT_incongruent_VP_mismatch_ conditions, using an FDR-corrected alpha threshold. Bayesian paired samples t-tests were also performed using a Cauchy prior of 0.707, zero-centred. To check for effects of hand disownership at the individual level in each condition, kinematic variables were also correlated with responses to S3, S9, S10, and S11. This was performed with Kendall rank correlations, using an FDR-corrected alpha threshold, along with Bayesian Kendall rank correlations (stretched beta prior width of 1). The same analysis was also performed for S4, as in experiment 1, to assess the effect of agency on kinematics.

### Results

#### Questionnaire

**Fig 4:**
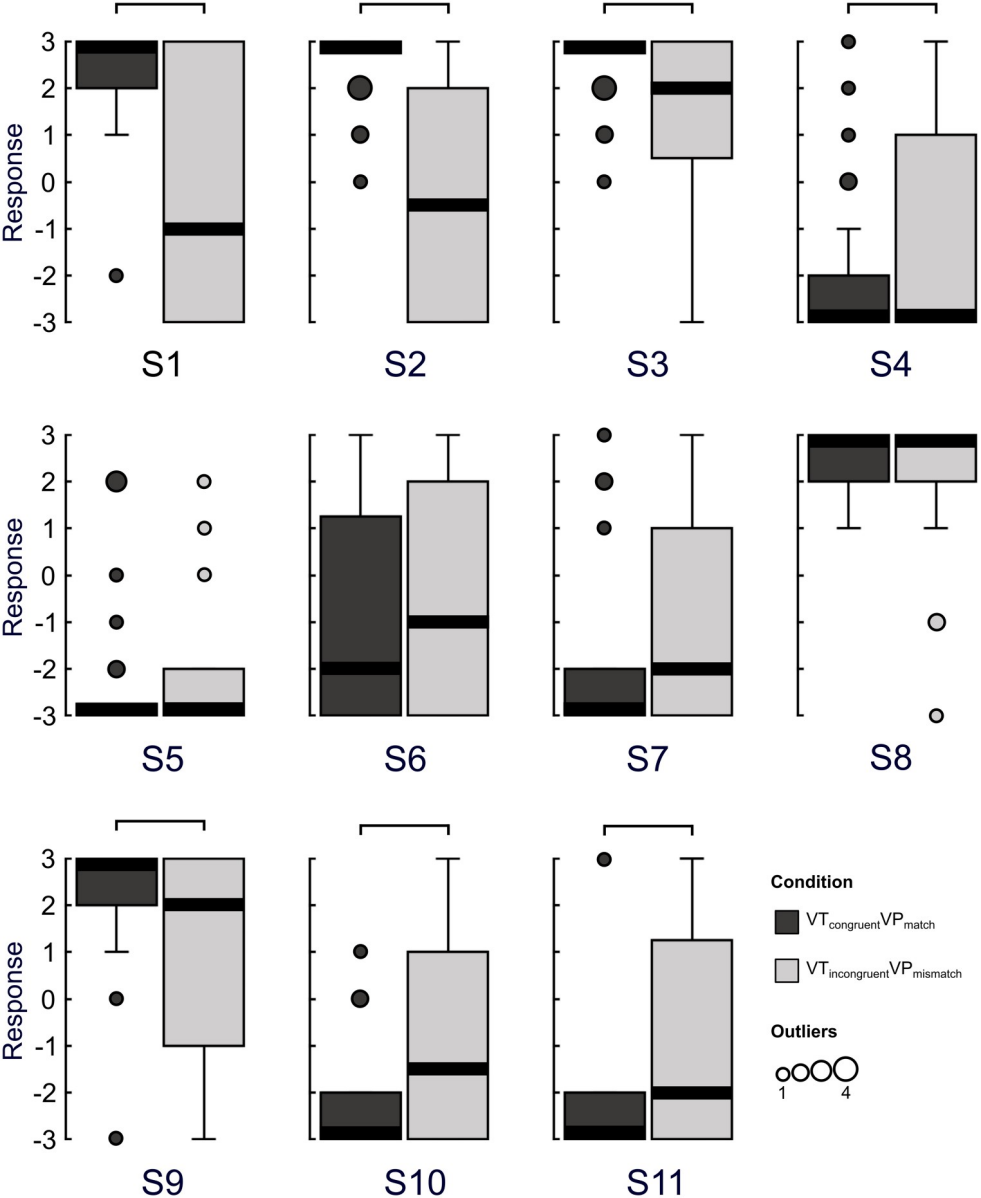
Experiment 2 questionnaire results. Subjective experience during two levels of multisensory (dis)integration was assessed by comparing agreement with questionnaire statements. Boxplots indicate group responses to each statement for each condition (-3 = strongly disagree, +3 = strongly agree). The solid black lines indicate the median. The number of outliers at any given response value are indicated by the size of the outlying point. The connections above the boxes indicate statistically significant differences between conditions (Wilcoxon signed-rank test, FDR-corrected, p≤.0101).

As in experiment 1, participants showed a reduced sense of ownership over their hand when visuotactile and visuoproprioceptive information were incongruent (Fig 4). This was reflected in FDR-corrected (p≤.0101) statistically significant differences between the VT_congruent_VP_match_ and VT_incongruent_VP_mismatch_ conditions in statements assessing limb (dis)ownership: S3 (Z = -3.11, p = .00190), S9 (Z = -3.50, p<.001), S10 (Z = -3.79, p<.001), and S11 (Z = -3.07, p = .00212). In line with our hypothesis, hand ownership as measured on S3 and S9 was lower and hand disownership as measured on S10 and S11 was higher in the condition with incongruent multisensory stimulation than the congruent condition. Importantly, there was no statistically significant difference between conditions in statement S8, assessing the degree to which the hand on the screen looked like the participant’s own hand (Z = -1.19, p = .236), which speaks against visual recognition of the hand as a significant factor for the condition-specific effects in our questionnaire data.

Participants showed a reduction in the perceptual binding of visual and tactile events on the hand in the VT_incongruent_VP_mismatch_ condition compared to the VT_congruent_VP_match_ condition, as reflected in a statistically significant difference between these conditions in the level of agreement to S2 (Z = -4.06, p<.001). In addition, participants felt less like their hand was located in the position shown on the screen (S1) in the VT_incongruent_VP_mismatch_ condition compared to the VT_congruent_VP_match_ condition (Z = -3.97, p<.001).

Responses to S4 (“It seemed like I was unable to move my hand”) received low scores in both conditions, but less so in the VT_incongruent_VP_mismatch_ condition (Z = -2.57, p = .0101), which suggested a greater reduction in perceived agency over the hand in this condition.

There was no statistically significant difference between the two conditions in the control statements S5 (Z = -0.157, p = .876), assessing the perception of the hand disappearing, or S6 (Z = -1.48, p = .139), assessing the sensation of numbness in the hand. With an FDR-corrected alpha threshold, there was no statistically significant difference in the level of agreement to the control statement S7, “I did not know exactly where my hand was located” (Z = -2.03, p = .0420).

#### Skin conductance response

There was no statistically significant difference in the skin conductance response amplitude between the VT_congruent_VP_match_ (mean±SE = 0.443±0.0736 μS) and VT_incongruent_VP_mismatch_ (0.390±0.0617 μS) conditions (t(27) = 1.04, p = .306, g_rm_ = 0.141, BF_10_ = 0.328, max BF_10_ = 1.00 at Cauchy prior width 0.0005). The Bayes factor suggested that, given the data, the null hypothesis was more likely than the alternative hypothesis (VT_congruent_VP_match_ ≠ VT_incongruent_VP_mismatch_). In addition, there were no statistically significant correlations between the skin conductance response and any of the questionnaire statements assessing hand (dis)ownership (|r_τ_|≤.095, p≥.515; Table H in S1 File), with greater support for a null hypothesis in all cases (BF_10_≤0.309, max BF_10_ = 1.00 at Cauchy prior width 0.0001 in all cases).

#### Kinematics

There were no statistically significant differences between the VT_congruent_VP_match_ and VT_incongruent_VP_mismatch_ conditions at the group level for any of the three kinematic variables: RT (mean±SE = 520±8.50 ms versus 526±9.45 ms, t(29) = 0.950, p = .350, g_rm_ = 0.120, BF_10_ = 0.294, max BF_10_ = 1.00 at Cauchy prior width 0.0005), PA (797±48.1 cm/s^2^ versus 790±42.1 cm/s^2^, t(29) = 0.231, p = .819, g_rm_ = 0.0255, BF_10_ = 0.199, max BF_10_ = 1.00 at Cauchy prior width 0.0005), and PV (27.6±1.58 cm/s versus 27.7±1.37 cm/s, t(29) = 0.203, p = .841, g_rm_ = 0.0186, BF_10_ = 0.198, max BF_10_ = 1.00 at Cauchy prior width 0.0005). The Bayesian analyses suggested more support for the null hypothesis than the alternative.

There were also no statistically significant correlations between kinematic variables and questionnaire statements assessing the subjective sensation of hand (dis)ownership (Tables I and J in S1 File). In all cases, |r_τ_|≤.200, p≥.155. In most cases, Bayesian analyses suggested greater support for the null hypothesis (0.235≤BF_10_≤0.751) than the alternative hypothesis, with the maximum BF_10_ = 1.31 at a stretched beta prior width of 0.102. Unlike experiment 1, we did not observe any evidence of a positive correlation between PA and the responses to S3 or between PV and the responses to S3 in the VT_incongruent_VP_mismatch_ condition.

As in experiment 1, there were no statistically significant FDR-corrected correlations between kinematic variables and the responses to S4 (Tables I and J in S1 File), assessing the sense of agency over the hand (r_τ_|≤.340, p≥.0164, 0.249≤BF_10_≤6.68). However, the Bayesian analysis did suggest support for a correlation between PV and the responses to S4 for both the VT_congruent_VP_match_ (r_τ_ = -.311, BF_10_ = 3.86, max BF_10_ = 4.72 at a stretched beta prior width of 0.360) and VT_incongruent_VP_mismatch_ (r_τ_ = -.340, BF_10_ = 6.68, max BF_10_ = 7.70 at a stretched beta prior width of 0.436) conditions, suggesting that participants who reported a reduced sense of agency for their hand tended to move their finger more slowly.

#### Post hoc assessment of the illusion-susceptible subset

To further verify the absence of an effect related to the subjective sensation of hand disownership, we performed an additional one-sided Bayesian analysis of only the participants who showed a reduction in perceived ownership, using the same prior distributions as described in experiment 1. We based this subset (n = 22) on participants who reported reduced ownership sensations in either S3, as in experiment 1, or S10, since the correlation between S10 and kinematic variables had the least convincing evidence for the null hypothesis. Reductions in ownership were defined as a more positive response to S3 in the VT_congruent_VP_match_ compared to VT_incongruent_VP_mismatch_ condition or vice versa for S10. Importantly, there was convincing evidence for a correlation between participant responses to these statements in the VT_incongruent_VP_mismatch_ condition (r_τ_ = -.460, p = .00209, BF_10_ = 107), suggesting that those who expressed a sense of disownership in their response to one statement were likely to express a sense of disownership in response to the other.

Comparing the VT_congruent_VP_match_ and VT_incongruent_VP_mismatch_ conditions highlighted that 59.1% of participants had an increased RT in the VT_incongruent_VP_mismatch_ condition. However, only 50% of participants showed a reduced PA, while 54.5% showed a reduced PV. The prior centred on 0.74 suggested greater support for the null hypothesis than the alternative hypothesis for PA (770±54.3 cm/s^2^ versus 738±47.3 cm/s^2^, BF_10_ = 0.199, g_rm_ = 0.127), PV (26.6±1.87 cm/s versus 26.9±1.56 cm/s^2^, BF_10_ = 0.139, g_rm_ = 0.0768), and RT (524±10.4 ms versus 530±10.8 ms, BF_10_ = 0.151, g_rm_ = 0.128). The prior centred on 0.37 did not provide support in either direction for PA (BF_10_ = 0.980), PV (BF_10_ = 0.794), or RT (BF_10_ = 0.835). The default Cauchy prior did not provide support in either direction for RT (BF_10_ = 0.467, max BF_10_ = 1.09 at Cauchy prior width 0.0494), PA (BF_10_ = 0.530, max BF_10_ = 1.15 at Cauchy prior width 0.0644), and PV (BF_10_ = 0.449, max BF_10_ = 1.08 at Cauchy prior width 0.0381). Overall these results suggested that, when assessing only the participants that reported a reduction in the subjective sensation of limb ownership, there was little support for a large (0.74) effect on kinematics. However, we could not draw strong conclusions regarding a smaller effect in experiment 2.

## Experiment 3 (control)

Whilst our two experiments both indicated that weakening the subjective sense of ownership over the hand did not interfere with basic movement, we did not account for the fact that questionnaire responses were recorded following passive observation of the hand, rather than the movement task in which our kinematic data were collected. That participants had to prepare to move following visuotactile stimulation might have altered the strength of the illusion, one may argue, if there exists a dynamic interplay between the motor system and body ownership (e.g., [74]). This might interfere with any potential effects of limb disownership on movement. Though we considered this unlikely, we aimed to refute this possibility with a control experiment. We assessed participant responses to statement S3 (“It felt as if I was looking at my own hand”) following passive observation of the hand during multisensory disintegration (as in experiments 1 and 2), as well as observation prior to rapid finger abduction.

### Methods

Unless otherwise stated, the equipment and methods were identical to experiment 2.

#### Participants

We recruited 24 new participants (mean±SD age = 24.8±3.01 years, 10 female, 1 left-handed). None had taken part in the previous experiments. The experiment was approved by the local ethics committee (ref: 2010/548-31/2, 2018/322, and 2018/2117), and participants provided written informed consent.

#### Procedure

The mean±SD size of the hand on the screen was approximately 90.6±7.57% of the actual length and 99.2±4.55% of the actual width. Participants took part in four conditions, which were tested in four counterbalanced blocks: passive VT_congruent_VP_match_, passive VT_incongruent_VP_mismatch_, moving VT_congruent_VP_match_, and moving VT_incongruent_VP_mismatch_. Each block consisted of three trials. In the passive conditions, participants were requested to simply observe their hand during the 30 second visuotactile stimulation period, following which the screen turned blue and they were verbally presented with statement S3 by the experimenter (whilst the screen remained blue). In the moving conditions, participants observed their hand during the 30 second visuotactile stimulation period, following which the screen turned blue and a tone played to signal them to rapidly abduct their index finger (as in the motion-tracking data collection component of experiment 2). They were then verbally presented with statement S3 by the experimenter, and responded whilst the screen remained blue.

The experimenter informed participants before each block whether they would be required to perform a movement following the 30 second periods of visuotactile stimulation. If putative movement preparation prevents a reduction in ownership during multisensory disintegration, then one would expect a greater level of agreement to statement S3 in the moving VT_incongruent_VP_mismatch_ condition compared to the passive VT_incongruent_VP_mismatch_ condition.

#### Data analysis

The median response to statement S3 for the three inductions was used to create a single value per condition for each participant. We then used Wilcoxon signed-rank tests to compare the difference between VT_congruent_VP_match_ and VT_incongruent_VP_mismatch_ in passive and moving conditions, the passive and moving VT_incongruent_VP_mismatch_ conditions, and the magnitude of the illusion effect (VT_congruent_VP_match_—VT_incongruent_VP_mismatch_) between passive and moving conditions.

### Results

**Fig 5:**
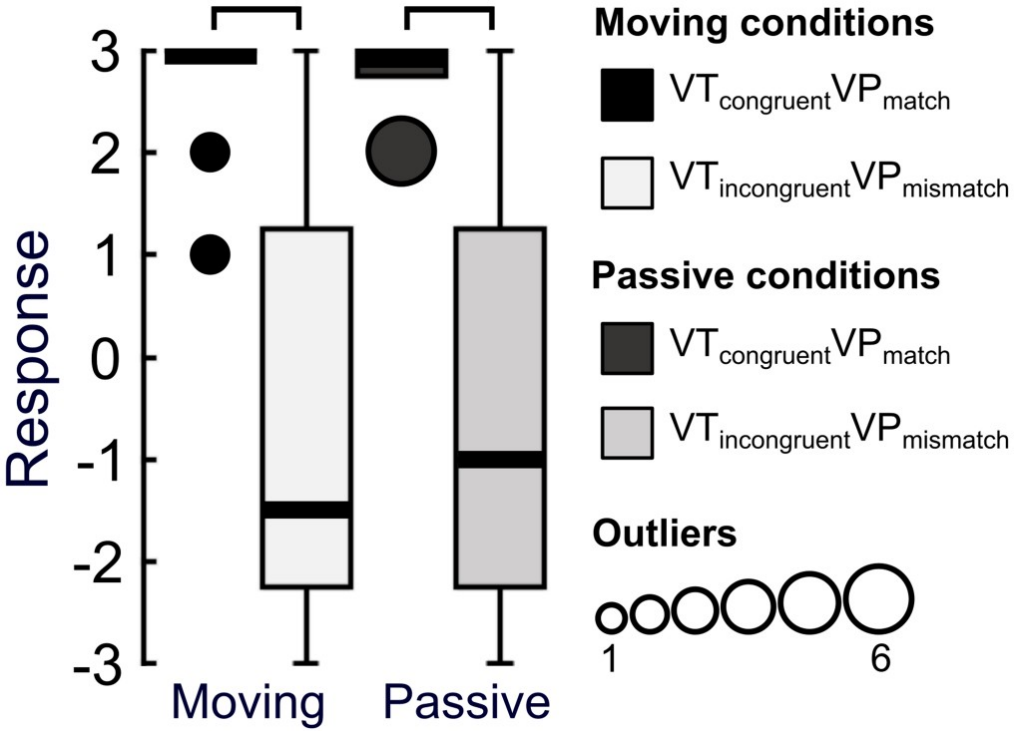
Experiment 3 responses to statement S3. Agreement with the statement “It felt as if I was looking at my own hand” was compared when participants were required to either move their index finger or remain passive following two levels of multisensory (dis)integration. Boxplots indicate group responses to each statement for each condition (-3 = strongly disagree, +3 = strongly agree). The solid black lines indicate the median. The number of outliers at any given response value are indicated by the size of the outlying point. The connections above the boxes indicate statistically significant differences between conditions (Wilcoxon signed-rank test, p<.001).

We observed that agreement with statement S3 was statistically significantly reduced in the VT_incongruent_VP_mismatch_ condition compared to the VT_congruent_VP_match_ condition in both moving (Z = -3.75, p<.001) and passive (Z = -3.85, p<.001) scenarios (Fig 5). There was no significant difference between the magnitude of illusion effect between passive and moving conditions (Z = -0.328, p = .743), or between agreement to S3 in the passive and moving VT_incongruent_VP_mismatch_ conditions (Z = -0.365, p = .715). Furthermore, the distribution of responses was highly similar between the moving and passive VT_incongruent_VP_mismatch_ conditions (Fig 5), suggesting that our multisensory disintegration paradigm was effective at reducing the sense of ownership over the hand, even when a movement had to be performed following visuotactile stimulation.

Since we seemed to observe greater reductions in ownership during multisensory disintegration in this experiment compared to experiments 1 and 2, possibly due to the different experimental design, we also decided post hoc to perform a Bayesian analysis on the motion-tracking data collected in the moving conditions (as in experiments 1 and 2). We suggest treating this analysis with caution, however, given the low number of trials collected and ultimately usable for each condition (65.3% of kinematic trials available following pre-processing).

We examined only the subset of participants who had at least one usable trial in both moving conditions, and that showed a reduced agreement with statement S3 in the moving VT_incongruent_VP_mismatch_ condition relative to the moving VT_congruent_VP_match_ condition (n = 15). The prior centred on 0.74 suggested greater support for the null hypothesis than the alternative hypothesis for PA (BF_10_ = 0.0207) and PV (BF_10_ = 0.00876), but not RT (BF_10_ = 0.918). The prior centred on 0.37 did not provide support in either direction for RT (BF_10_ = 1.92), but provided support in favour of the null hypothesis for PA (BF_10_ = 0.236) and PV (BF_10_ = 0.152). The default Cauchy prior did not provide support in either direction for RT (BF_10_ = 0.956), but provided support for the null hypothesis for PA (BF_10_ = 0.182) and PV (BF_10_ = 0.136). Overall these results suggested that, when assessing only the participants that reported a reduction in the subjective sensation of limb ownership, there was little support for effects on PA and PV. However, we could not draw strong conclusions regarding an effect on RT.

## General discussion

We examined whether experimentally induced reductions in the sensation of ownership over the hand would interfere with rapid finger movements. As shown in previous studies [15,48–54], the disintegration of multisensory information from the hand resulted in a reduction in the subjective sensation of ownership. We replicated this finding in three experiments through the manipulation of visuotactile and visuoproprioceptive information, and built on previous work by examining the reaction time, acceleration, and velocity of rapid finger movements following multisensory disintegration. Notably, we observed no statistically significant effect of reduced hand ownership on kinematic variables, either at the group or individual level, in two separate experiments. Bayesian analyses suggested that there was little evidence in favour of an effect, even when testing explicit hypotheses in only the participants that experienced a reduction in the subjective sensation of hand ownership. A control study confirmed that it was possible to reduce the sensation of ownership over the hand even when movement was required following illusion induction. Taken together, our results speak against a functional role of subjectively experienced body ownership in the motor system’s basic capacity to produce movement.

### Limb (dis)ownership and movement generation

After testing the effects of the rubber hand illusion on corticospinal excitability, della Gatta *et al*. [28] suggested that “if I believe that the hand is mine, then I must be ready to use it; if not, then the activity of the motor system is accordingly down-regulated” (p. 8). With this in mind, we expected that reducing the sensation of ownership over the real hand, regardless of the presence of a rubber hand, would negatively influence rapid finger movements. However, our results appear to be inconsistent with this idea. Why might this be? One possibility is that a strong sense of ownership over the body is not necessary for basic movement. This may be supported by a striking case study describing a patient with a sense of disownership for his entire body, following damage to the right temporoparietal cortex [75]. Despite this sensation of disownership, the patient apparently had no problems with motor planning or execution. In addition, an article by Osumi *et al*. [52] reported that delayed visual feedback during movement decreased muscle activity and movement speed as well as weakening the sense of ownership over the limb, but these variables were not correlated. These results provide some evidence beyond our experiment that a reduced sense of ownership over the body may not interfere with simple motor performance. This supports the idea that, although active or passive movement may play a role in generating a sense of ownership over the body [19–24], it is possible that this interaction is unidirectional.

However, that is not to say that body ownership is unimportant for guiding *action*, especially given that interactions with the world should be contingent on an accurate distinction between body and world. For example, the perceived size of our own body influences our perception of the size of objects [17], and the perceived position or extent of our own body in space influences how we interact with external targets [12,13,15]. Furthermore, body ownership may play a role in updating internal representations of the sensory state of the body, assisting in the generation of predictions about the consequences of our actions [76]. It may be that body ownership guides movement only when one must distinguish between which parts of the environment are bodily and which parts are not (i.e., during object-directed or defensive action [77]).

We might also consider that an effect of hand disownership sensations on rapid finger movement does exist but is considerably smaller than the effect sizes used for our informed Bayesian analyses [28]. The fact that our examination of an illusion-susceptible subset in experiment 2 did not indicate support for the null hypothesis at a smaller effect size might support this (though, given the smaller sample size, which was used in anticipation of a stronger illusion effect, it is possible that our analysis was insensitive). We cannot exclude the possibility that reductions in the subjective sensation of ownership could induce small effects in movement kinematics, although it is worth considering whether these effects are large enough to be considered functionally meaningful. Indeed, the fact that we did not observe effects in the predicted direction in at least two thirds of susceptible individuals suggests little consistency in participant behaviour. On the other hand, the fact that very few participants explicitly rejected ownership of their own hand (i.e., gave negative ratings) in experiment 1 and 2, rather showing instead a reduction in ownership, may have influenced our likelihood of detecting kinematic effects. Given that individuals can visually recognise their own hand on the screen, it is possible that there is a limit to the extent that individuals will agree with disownership statements, regardless of the level of multisensory incongruence. However, we did observe greater reductions in the feeling of ownership over the hand in experiment 3, possibly because participants were more attentive when reporting their subjective experience, compared to experiments 1 and 2 where the questionnaire was presented at the end of the experiment. Notably, we observed similar null results for peak acceleration and velocity in this control study, though the low number of motion-tracking trials collected per condition suggests treating this analysis with caution.

Another explanation for our result is that reductions in hand ownership are disregarded when movement is necessary. This might provide an explanation for the discrepancy between our findings and those reported in studies of body ownership recording MEPs [28,31–33]. It is possible that motor behaviour is subject to top-down control in our paradigm, ensuring successful movement even when the sensation of ownership over the limb is reduced. Alternatively, the sense of agency, which co-occurs with ownership under normal conditions and is sometimes affected by manipulations of ownership ([5,21,78,79], but see [19] for a dissociation of agency and ownership), may be the key component in mediating the capacity of the motor system. Interestingly, we observed in experiment 2 that individuals who reported a greater loss of agency for their own hand moved their finger more slowly (i.e., with smaller velocity). We urge caution in drawing conclusions from this result, given that it was only observed in one experiment, and was not the focus of this experiment. However, this effect would be in keeping with suggestions that the sense of agency and the activity of the sensorimotor system are closely linked (e.g.,[32,34,54,67]).

### Experimental limitations

Given our claims in favour of a null hypothesis, it is essential to consider whether our experimental approach could have resulted in failure to detect an existing effect. We must consider the general lack of changes in skin conductance response to threat, our objective measure of changes in body ownership, following multisensory disintegration. Whilst this is in keeping with another recent study using a multisensory disintegration paradigm [53], it is in contrast to Gentile *et al*. [48], who observed reductions in skin conductance response to threat in conditions inducing reductions in the sensation of own hand ownership. It is possible that this stems from differences between our paradigm and the one employed by Gentile *et al*. [48]. We can identify three key differences with respect to the present study.

First, in their experiment, participants looked at a pre-recorded video of their hand laying on a table through a set of head-mounted displays, whilst their real hand (positioned congruently or incongruently) was stroked in sync or out of sync with the video. Importantly, in their incongruent visuoproprioceptive conditions, the participants put their real hand on their chest, far away from the hand image they were looking at, which still appeared to be laying outstretched on the table. This gross visuoproprioceptive incongruency could have boosted disownership of the hand in view and led to stronger reductions in threat-evoked skin conductance response than in the present study. In our experiment, participants were always aware that their real hand was inside the box and being approached with the knife, regardless of condition, and this could have led to reduced differences in skin conductance response between conditions. Second, since Gentile *et al*. used pre-recorded videos, they could present a more ‘dramatic’ threat stimuli than we could in present in a live scenario in which we had to be very careful on every trial not to touch the participant’s real hand with the (blunted) knife. Third, in the present hand illusion box setup, the match between the seen and felt orientation of the hand is near-perfect when visuoproprioceptive stimuli are matched; as such, there exists strong visuoproprioceptive congruency that may boost hand ownership and make it harder to achieve strong disownership effects even when visuotactile asynchrony is applied. In the fMRI-based experiment by Gentile *et al*., it was not possible to achieve a perfect match between the pre-recorded movie of the hand and the position of the participant’s real hand in the MRI scanner environment. Thus, we speculate that subtle visuoproprioceptive incongruencies in their congruent condition could have augmented the effect of visuotactile synchrony and made it easier to achieve a strong disownership effect with the asynchrony manipulation. With these factors in mind, it is possible that our multisensory disintegration paradigm resulted in both subjective and objective reductions in body ownership, but that the experimental setup made the measurement of objective changes in the sense of body ownership ineffective. However, we cannot exclude the possibility that limb disownership does in fact interfere with the control of the limb but may be reliant on concomitant autonomic change, which did not occur in our experiment (possibly because participants appeared to show a reduction in the sense of ownership following multisensory disintegration, rather than outright rejection). The relative lack of studies examining hand disownership, compared to the rubber hand illusion, means that there is much more to be done to quantify the objective elements of this illusory state.

Related to this, we are aware that there is a lack of established experimental protocols for assessing the subjective sensation of own limb disownership, unlike the rubber hand illusion where specific questionnaire items are often used (e.g., [3,5]). There is some correspondence between our questionnaire responses and those resulting from similar statements in a study performed by a different group [50]. For example, whilst we used statement S7 (“I did not know exactly where my hand was located”) as a control, Kannape *et al*. [50] used a similar statement as a disownership measure, with some of their data suggesting an increased agreement with this statement following multisensory disintegration. We believed that this question would assess explicit knowledge regarding veridical location of the hand, which did not change across conditions. However, we observed reduced disagreement to this statement following multisensory disintegration in experiment 1, suggesting that the statement may have instead captured sensory uncertainty, concomitant with a reduction in the subjective sensation of ownership. Similarly, we used statement S5, addressing the perception of the hand disappearing, as a control statement. We did not expect participants to report agreement with this statement, given the near constant view of their own hand on the screen, and indeed the median response to this statement in all conditions in experiments 1 and 2 suggested that participants strongly disagreed with it. However, other research has reported positive responses to a similar statement following the disintegration of multisensory feedback of the hand, albeit in different experimental contexts [49,50]. We must consider that different experimental protocols will afford different multisensory experiences. For example, some multisensory disintegration experiments manipulated online passive multisensory feedback of the real body as reported here [50,53,54], though without gross visuoproprioceptive conflict. Another used pre-recorded videos [48]. It appears that further work will be necessary to develop a consensus for appropriate subjective assessments of own limb disownership, and how they might be influenced by the choice of experimental paradigm.

Another potential limitation is the time delay (1.5 to 2.5 seconds in experiment 1, 0.5 to 1 second in experiment 2 and 3, following experimenter trigger) between the end of hand observation and the cue to movement. These delays may have been too long, such that any downregulation of the motor system could have ‘worn off’ by the cue to movement. Although we have no data on the duration of the after-effect of body disownership illusions, we know that the after-effects on limb ownership illusions [80] and full-body ownership illusions are quite long-lived and can produce behavioural effects for many seconds after multisensory stimulation has stopped and the artificial body is no longer in view [18,81,82]. Lastly, it is also possible that the duration of visuotactile stimulation (13.5 seconds in experiment 1, 30 seconds in experiment 2 and 3) may have been too short to induce strong effects on later finger movement. However, the classic rubber hand illusion tends to occur in less than 30 seconds [66,83,84], and previous work using a multisensory disintegration paradigm confirms that illusion induction can indeed occur after 13.5 seconds [48].

### Conclusion

Our results suggest that reducing the subjective sensation of own hand ownership does not seem to interfere with rapid finger movement, at least in the paradigm reported here. Changes in the excitability of the motor system previously observed in body ownership illusions may not have functional consequences, or could be moderated by top-down processes in cases where movement is actually required. Alternatively, a stronger and more complete disownership of the hand than we were able to achieve in the present paradigm may be a requirement to interfere with movement. Future studies should aim to shed more light on the interactions between basic movement and the excitability of the motor system as they relate to body ownership, preferably trying to establish common ground across different types of body illusion.

## Supporting information

S1 File Detailed results. (PDF)

S2 File Source image for Fig 1 that was adapted with permission from Abdulkarim & Ehrsson (2018). (TIFF)
