# Supplementary material for: Weakening the subjective sensation of own hand ownership does not interfere with rapid finger movements
Source: PLoS One. 2019 Oct 4;14(10):e0223580. doi: 10.1371/journal.pone.0223580 (PMC6777829; doi:10.1371/journal.pone.0223580)
Supplement: S1 File — (PDF) [file pone.0223580.s001.pdf]

S1 Supporting Information: Detailed results

Table A: Experiment 1 questionnaire analysis (Wilcoxon signed-rank)

Statement	Compared conditions		Z	p
S1	$VT_{congruent}VP_{match}$	$VT_{incongruent}VP_{match}$	-3.13	.00175
	$VT_{congruent}VP_{match}$	$VT_{congruent}VP_{mismatch}$	-4.87	.00000114
	$VT_{congruent}VP_{match}$	$VT_{incongruent}VP_{mismatch}$	-5.43	.0000000560
	$VT_{incongruent}VP_{match}$	$VT_{congruent}VP_{mismatch}$	-3.04	.00235
	$VT_{incongruent}VP_{match}$	$VT_{incongruent}VP_{mismatch}$	-4.85	.00000127
	$VT_{congruent}VP_{mismatch}$	$VT_{incongruent}VP_{mismatch}$	-2.90	.00374
S2	$VT_{congruent}VP_{match}$	$VT_{incongruent}VP_{match}$	-4.51	.00000664
	$VT_{congruent}VP_{match}$	$VT_{congruent}VP_{mismatch}$	-1.31	.189
	$VT_{congruent}VP_{match}$	$VT_{incongruent}VP_{mismatch}$	-4.87	.00000111
	$VT_{incongruent}VP_{match}$	$VT_{congruent}VP_{mismatch}$	-4.87	.00000110
	$VT_{incongruent}VP_{match}$	$VT_{incongruent}VP_{mismatch}$	-2.94	.00329
	$VT_{congruent}VP_{mismatch}$	$VT_{incongruent}VP_{mismatch}$	-5.25	.000000153
S3	$VT_{congruent}VP_{match}$	$VT_{incongruent}VP_{match}$	-2.85	.00436
	$VT_{congruent}VP_{match}$	$VT_{congruent}VP_{mismatch}$	-2.36	.0184
	$VT_{congruent}VP_{match}$	$VT_{incongruent}VP_{mismatch}$	-4.80	.00000162
	$VT_{incongruent}VP_{match}$	$VT_{congruent}VP_{mismatch}$	-0.390	.697
	$VT_{incongruent}VP_{match}$	$VT_{incongruent}VP_{mismatch}$	-3.83	.000128
	$VT_{congruent}VP_{mismatch}$	$VT_{incongruent}VP_{mismatch}$	-4.15	.0000338
S4	$VT_{congruent}VP_{match}$	$VT_{incongruent}VP_{match}$	-3.07	.00217
	$VT_{congruent}VP_{match}$	$VT_{congruent}VP_{mismatch}$	-1.17	.244
	$VT_{congruent}VP_{match}$	$VT_{incongruent}VP_{mismatch}$	-2.50	.0125
	$VT_{incongruent}VP_{match}$	$VT_{congruent}VP_{mismatch}$	-2.82	.00488
	$VT_{incongruent}VP_{match}$	$VT_{incongruent}VP_{mismatch}$	-0.378	.705
	$VT_{congruent}VP_{mismatch}$	$VT_{incongruent}VP_{mismatch}$	-2.62	.00893
S5	$VT_{congruent}VP_{match}$	$VT_{incongruent}VP_{match}$	-0.543	.587
	$VT_{congruent}VP_{match}$	$VT_{congruent}VP_{mismatch}$	-1.03	.303
	$VT_{congruent}VP_{match}$	$VT_{incongruent}VP_{mismatch}$	-1.62	.106
	$VT_{incongruent}VP_{match}$	$VT_{congruent}VP_{mismatch}$	-0.900	.368
	$VT_{incongruent}VP_{match}$	$VT_{incongruent}VP_{mismatch}$	-1.61	.107
	$VT_{congruent}VP_{mismatch}$	$VT_{incongruent}VP_{mismatch}$	-0.897	.369
S6	$VT_{congruent}VP_{match}$	$VT_{incongruent}VP_{match}$	-2.21	.0270
	$VT_{congruent}VP_{match}$	$VT_{congruent}VP_{mismatch}$	-0.358	.720
	$VT_{congruent}VP_{match}$	$VT_{incongruent}VP_{mismatch}$	-2.92	.00354
	$VT_{incongruent}VP_{match}$	$VT_{congruent}VP_{mismatch}$	-2.64	.00837
	$VT_{incongruent}VP_{match}$	$VT_{incongruent}VP_{mismatch}$	-1.03	.302
	$VT_{congruent}VP_{mismatch}$	$VT_{incongruent}VP_{mismatch}$	-3.20	.00140
S7	$VT_{congruent}VP_{match}$	$VT_{incongruent}VP_{match}$	-2.52	.0118
	$VT_{congruent}VP_{match}$	$VT_{congruent}VP_{mismatch}$	-3.62	.000296
	$VT_{congruent}VP_{match}$	$VT_{incongruent}VP_{mismatch}$	-4.41	.0000103
	$VT_{incongruent}VP_{match}$	$VT_{congruent}VP_{mismatch}$	-2.07	.0381
	$VT_{incongruent}VP_{match}$	$VT_{incongruent}VP_{mismatch}$	-3.15	.00166
	$VT_{congruent}VP_{mismatch}$	$VT_{incongruent}VP_{mismatch}$	-1.29	.197

Table B: Experiment 1 correlations between responses to S3 and SCR to threat

Condition	r_τ	p	BF_{10}	Bayes factor robustness	
				Max BF_{10}	Stretched beta prior width
$VT_{congruent}VP_{match}$	-.288	.156	1.04	1.39	0.227
$VT_{incongruent}VP_{match}$	-.167	.392	0.464	1.00	0.0001
$VT_{congruent}VP_{mismatch}$	.080	.684	0.339	1.00	0.0001
$VT_{incongruent}VP_{mismatch}$	-.307	.101	1.22	1.56	0.257

Table C: Experiment 1 reaction time analysis

Two-tailed paired samples t-tests and two-sided paired samples Bayesian t-tests; mean±SE values for $VT_{congruent}VP_{match}$ (500±7.11ms), $VT_{incongruent}VP_{match}$ (496±7.56ms), $VT_{congruent}VP_{mismatch}$ (502±7.23ms), $VT_{incongruent}VP_{mismatch}$ (499±7.45ms).

Compared conditions		t(47)	p	BF_{10}	Bayes factor robustness	
					Max BF_{10}	Cauchy prior width
$VT_{congruent}VP_{match}$	$VT_{incongruent}VP_{match}$	0.997	.324	0.250	1.00	0.0005
$VT_{congruent}VP_{match}$	$VT_{congruent}VP_{mismatch}$	-0.398	.692	0.169	1.00	0.0005
$VT_{congruent}VP_{match}$	$VT_{incongruent}VP_{mismatch}$	0.074	.942	0.157	1.00	0.0005
$VT_{incongruent}VP_{match}$	$VT_{congruent}VP_{mismatch}$	-1.30	.201	0.343	1.00	0.0005
$VT_{incongruent}VP_{match}$	$VT_{incongruent}VP_{mismatch}$	-0.843	.404	0.219	1.00	0.0005
$VT_{congruent}VP_{mismatch}$	$VT_{incongruent}VP_{mismatch}$	0.447	.657	0.172	1.00	0.0005

Table D: Experiment 1 peak acceleration analysis

Two-tailed paired samples t-tests and two-sided paired samples Bayesian t-tests; mean±SE values for $VT_{congruent}VP_{match}$ (855±46.2cm/s^2), $VT_{incongruent}VP_{match}$ (863±50.7cm/s^2), $VT_{congruent}VP_{mismatch}$ (861±45.6cm/s^2), $VT_{incongruent}VP_{mismatch}$ (857±51.7cm/s^2).

Compared conditions		t(47)	p	BF_{10}	Bayes factor robustness	
					Max BF_{10}	Cauchy prior width
$VT_{congruent}VP_{match}$	$VT_{incongruent}VP_{match}$	-0.267	.791	0.162	1.00	0.0005
$VT_{congruent}VP_{match}$	$VT_{congruent}VP_{mismatch}$	-0.228	.820	0.161	1.00	0.0005
$VT_{congruent}VP_{match}$	$VT_{incongruent}VP_{mismatch}$	-0.078	.938	0.157	1.00	0.0005
$VT_{incongruent}VP_{match}$	$VT_{congruent}VP_{mismatch}$	0.053	.958	0.157	1.00	0.0005
$VT_{incongruent}VP_{match}$	$VT_{incongruent}VP_{mismatch}$	0.232	.818	0.161	1.00	0.0005
$VT_{congruent}VP_{mismatch}$	$VT_{incongruent}VP_{mismatch}$	0.134	.894	0.158	1.00	0.0005

Table E: Experiment 1 peak velocity analysis

Two-tailed paired samples t-tests and two-sided paired samples Bayesian t-tests; mean±SE values for $VT_{congruent}VP_{match}$ (33.4±1.83cm/s), $VT_{incongruent}VP_{match}$ (34.0±1.90cm/s), $VT_{congruent}VP_{mismatch}$ (33.7±1.85cm/s), $VT_{incongruent}VP_{mismatch}$ (33.4±1.99cm/s).

Compared conditions		t(47)	p	BF_{10}	Bayes factor robustness	
					Max BF_{10}	Cauchy prior width
$VT_{congruent}VP_{match}$	$VT_{incongruent}VP_{match}$	-0.592	.557	0.185	1.00	0.0005
$VT_{congruent}VP_{match}$	$VT_{congruent}VP_{mismatch}$	-0.338	.737	0.166	1.00	0.0005
$VT_{congruent}VP_{match}$	$VT_{incongruent}VP_{mismatch}$	-0.014	.989	0.157	1.00	0.0005
$VT_{incongruent}VP_{match}$	$VT_{congruent}VP_{mismatch}$	0.284	.778	0.163	1.00	0.0005
$VT_{incongruent}VP_{match}$	$VT_{incongruent}VP_{mismatch}$	0.690	.494	0.196	1.00	0.0005
$VT_{congruent}VP_{mismatch}$	$VT_{incongruent}VP_{mismatch}$	0.275	.785	0.163	1.00	0.0005

Table F: Experiment 1 correlations between responses to S3 (ownership) and kinematic variables

	RT					PA					PV				
	r_τ	p	BF_{10}	Bayes factor robustness		r_τ	p	BF_{10}	Bayes factor robustness		r_τ	p	BF_{10}	Bayes factor robustness	
				Max BF_{10}	Stretched beta prior width				Max BF_{10}	Stretched beta prior width				Max BF_{10}	Stretched beta prior width
**$VT_{congruent}VP_{match}$**	-.025	.832	0.193	1.00	0.0001	-.083	.477	0.264	1.00	0.0001	-.054	.648	0.216	1.00	0.0001
**$VT_{incongruent}VP_{match}$**	-.174	.119	0.826	1.59	0.102	.010	.931	0.188	1.00	0.0001	.097	.384	0.297	1.00	0.0001
**$VT_{congruent}VP_{mismatch}$**	.214	.057	1.76	2.83	0.171	-.032	.774	0.197	1.00	0.0001	-.003	.976	0.187	1.00	0.0001
**$VT_{incongruent}VP_{mismatch}$**	.094	.375	0.290	1.00	0.0001	.220	.039	2.00	3.14	0.171	.227	.033	2.36	3.61	0.198

Table G: Experiment 1 correlations between responses to S4 (agency) and kinematic variables

	RT					PA					PV				
	r_τ	p	BF_{10}	Bayes factor robustness		r_τ	p	BF_{10}	Bayes factor robustness		r_τ	p	BF_{10}	Bayes factor robustness	
				Max BF_{10}	Stretched beta prior width				Max BF_{10}	Stretched beta prior width				Max BF_{10}	Stretched beta prior width
**$VT_{congruent}VP_{match}$**	-.016	.889	0.190	1.00	0.0001	.006	.957	0.188	1.00	0.0001	-.011	.924	0.188	1.00	0.0001
**$VT_{incongruent}VP_{match}$**	-.021	.851	0.191	1.00	0.0001	.097	.384	0.298	1.00	0.0001	.089	.429	0.275	1.00	0.0001
**$VT_{congruent}VP_{mismatch}$**	-.214	.055	1.78	2.86	0.171	.108	.332	0.333	1.01	0.0092	.059	.597	0.222	1.00	0.0001
**$VT_{incongruent}VP_{mismatch}$**	-.069	.528	0.237	1.00	0.0001	.085	.433	0.269	1.00	0.0001	.082	.456	0.260	1.00	0.0001

Table H: Experiment 2 SCR-questionnaire correlations

Statement	$VT_{congruent}VP_{match}$					$VT_{incongruent}VP_{mismatch}$				
				Bayes factor robustness					Bayes factor robustness	
	r_τ	p	BF_{10}	Max BF_{10}	Stretched beta prior width	r_τ	p	BF_{10}	Max BF_{10}	Stretched beta prior width
S3	-.065	.677	0.272	1.00	0.0001	-.095	.515	0.309	1.00	0.0001
S9	-.081	.597	0.290	1.00	0.0001	-.081	.570	0.290	1.00	0.0001
S10	.093	.547	0.307	1.00	0.0001	-.009	.952	0.244	1.00	0.0001
S11	-.054	.732	0.263	1.00	0.0001	-.088	.540	0.300	1.00	0.0001

Table I: Experiment 2 kinematic-questionnaire correlations for $VT_{congruent}VP_{match}$

Statement		RT					PA					PV				
					Bayes factor robustness					Bayes factor robustness					Bayes factor robustness	
		r_τ	p	BF_{10}	Max BF_{10}	Stretched beta prior width	r_τ	p	BF_{10}	Max BF_{10}	Stretched beta prior width	r_τ	p	BF_{10}	Max BF_{10}	Stretched beta prior width
Ownership	S3	-.011	.942	0.236	1.00	0.0001	.062	.680	0.263	1.00	0.0001	.011	.942	0.236	1.00	0.0001
	S9	-.086	.561	0.291	1.00	0.0001	-.079	.591	0.282	1.00	0.0001	-.136	.355	0.403	1.00	0.0092
	S10	.114	.445	0.343	1.00	0.0001	-.059	.694	0.260	1.00	0.0001	-.017	.908	0.237	1.00	0.0001
	S11	-.103	.499	0.320	1.00	0.0001	-.089	.560	0.295	1.00	0.0001	-.060	.692	0.261	1.00	0.0001
Agency	S4	-.218	.134	0.928	1.50	0.146	-.242	.095	1.29	1.90	0.198	-.311	.032	3.86	4.72	0.360

Table J: Experiment 2 kinematic-questionnaire correlations for $VT_{incongruent}VP_{mismatch}$

Statement		RT					PA					PV				
					Bayes factor robustness					Bayes factor robustness					Bayes factor robustness	
		r_τ	p	BF_{10}	Max BF_{10}	Stretched beta prior width	r_τ	p	BF_{10}	Max BF_{10}	Stretched beta prior width	r_τ	p	BF_{10}	Max BF_{10}	Stretched beta prior width
Ownership	S3	.011	.939	0.236	1.00	0.0001	-.200	.155	0.751	1.31	0.102	-.157	.263	0.483	1.05	0.0369
	S9	-.185	.180	0.636	1.19	0.0827	-.111	.419	0.337	1.00	0.0001	-.106	.441	0.327	1.00	0.0001
	S10	.167	.222	0.529	1.09	0.0501	-.115	.402	0.345	1.00	0.0001	-.150	.274	0.450	1.03	0.0256
	S11	.060	.669	0.261	1.00	0.0001	-.026	.853	0.240	1.00	0.0001	0	1	0.235	1.00	0.0001
Agency	S4	-.044	.755	0.249	1.00	0.0001	-.263	.064	1.74	2.40	0.227	-.340	.016	6.68	7.70	0.436
