# Supplementary figures and images for: Weakening the subjective sensation of own hand ownership does not interfere with rapid finger movements
Source: PLoS One. 2019 Oct 4;14(10):e0223580. doi: 10.1371/journal.pone.0223580 (PMC6777829; doi:10.1371/journal.pone.0223580)

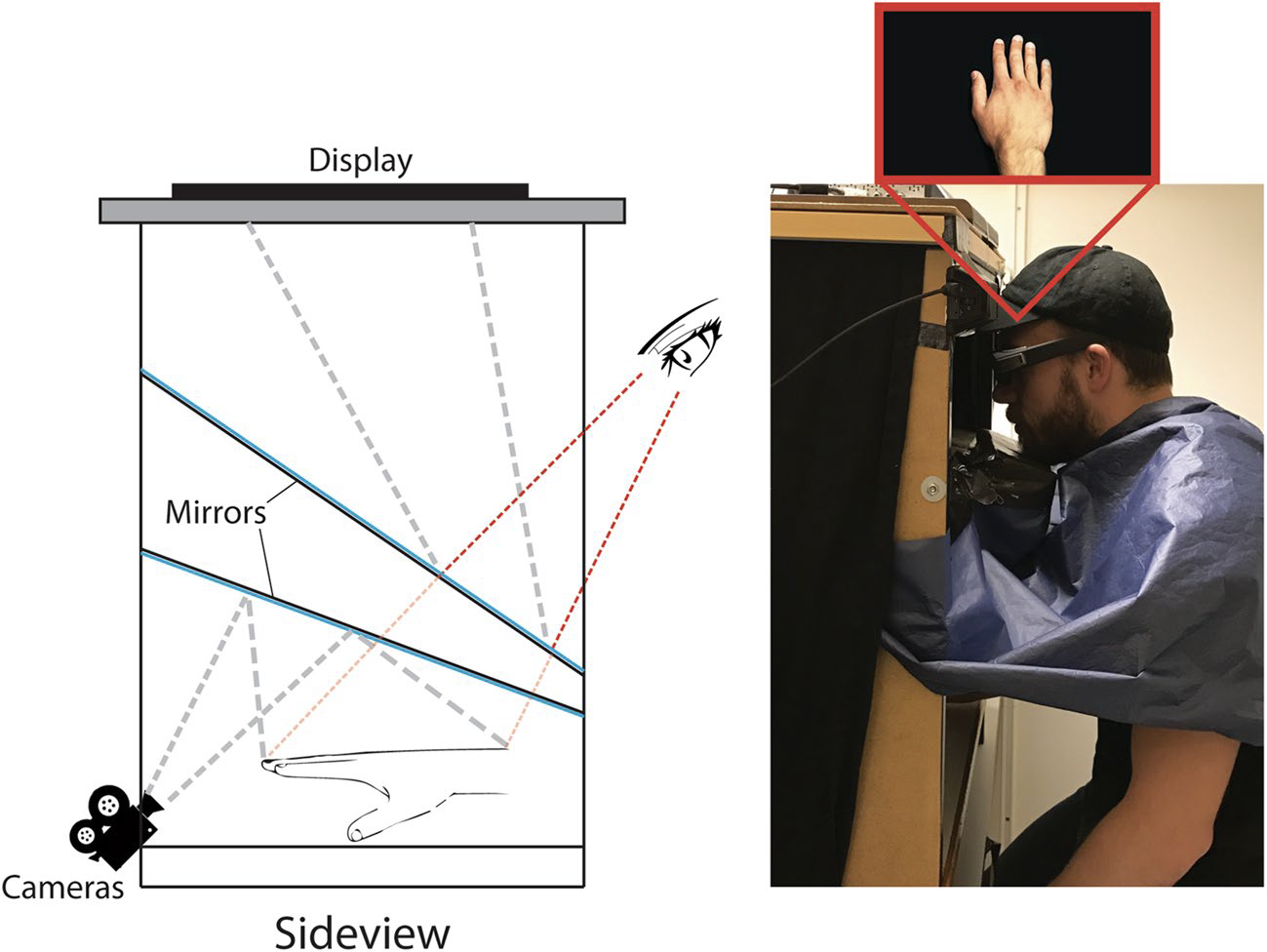

Supplement: S2 File — (TIFF) [file pone.0223580.s002.tiff]
